# The New Sydenham Society

**Published:** 1866-08

**Authors:** 


### The New Sydenham Society.

The Council of the New Sydenham Society begs to ask the attention of those who have not yet joined it to the special advantages which the current year offers to new members. *With this year it may almost be considered that a new series of works will be commenced.* The "Atlas of Skin Diseases" furnishes the only exception to this statement, and of this the former Fasciculi may be purchased without subscribing for the other works. The "Yearbook" has been wholly altered in plan and will be replaced by a volume of "Biennial Retrospects," the first of which will appear early in 1867. The Council has the pleasure to announce that these "Retrospects" will have the advantage of editorship as follows:

Medicine, Dr. Murchison; Surgery, (general,) Mr. T. Holmes; Ophthalmic Medicine and Surgery, Mr. T. Windsor; Midwifery, Dr. Barnes; Forensic Medicine, Toxicology and Hygiene, Dr. Hilton Fagge; Physiology, Mr. H. Power.

The following are among the Works which are now in preparation:

*Griesenger's Manual of Mental Diseases*, translated by Dr. Lockhart Robertson.

*Bernutz and Goupil on some of the Diseases of Women*, edited by Dr. Meadows.

*Hebra on Exanthems and diseases of the Skin*, translated by Dr. Hilton Fagge.

*Vogel on Diseases of the Kidney.*

